# Design and Analysis of a Magnetic Anchored and Cable-Driven Surgical Forceps for Minimally Invasive Surgery

**DOI:** 10.3390/mi16101109

**Published:** 2025-09-29

**Authors:** Jingwu Li, Yingtian Li, Zhongqing Sun, Zhijun Sun

**Affiliations:** 1 State Key Laboratory of Mechanics and Control for Aerospace Structures, Nanjing University of Aeronautics and Astronautics, Nanjing 210016, China; 2 Shenzhen Institute of Advanced Technology, Chinese Academy of Sciences, Shenzhen 518055, China

**Keywords:** magnetic surgical forceps, cable-driven, manipulator, control system

## Abstract

Magnetic surgical instruments are primarily driven by magnetic force and/or micro-motors. When micro-motors are used to drive motion, they are typically installed near the manipulator joints, resulting in a larger manipulator size due to the presence of micro-motors. We designed a magnetic anchored and cable-driven surgical forceps, which separates micro-motors from the manipulator through cables. The cables are responsible for transmitting motion and force from micro-motors to the manipulator. This design enables the integration of relatively large motors (diameter: 8 mm) while maintaining a compact overall diameter of the manipulator (diameter: 10 mm). This is beneficial for improving the flexibility of the manipulator and facilitating the coordination between surgical instruments. The manipulator of the magnetic anchored and cable-driven surgical forceps has three degrees of freedom (DoFs): pitch, yaw and clamping. A magnetic attraction experiment was conducted to measure the magnetic force on the magnetic surgical forceps with the variation of abdominal skin thickness. The results indicate that at a distance of 20 mm, the magnetic force exerted on the magnetic surgical forceps is 5.86 N, with a maximum vertical load capacity of 5.13 N. Additionally, an ex vivo experiment was conducted to validate the practicality of the magnetic anchored and cable-driven surgical forceps prototype.

## 1. Introduction

Compared to minimally invasive surgeries that need to cut several holes in the abdominal skin, single-port access surgery only needs to cut one incision with a diameter of 25–40 mm [1] to insert all of the surgical instruments. This further reduces the damage to the human body caused by the surgery, with a lower risk of postoperative complications and a better aesthetic effect. But the surgical instruments of single-port surgical robots are all connected to the machine placed outside of the abdominal cavity via rigid body linkages [2], causing the connecting parts of surgical instruments to be concentrated at the entry port during operations. The introduction of the magnetic anchoring and guidance system (MAGS) could effectively resolve the problems faced by single-port surgical robots [3]. That is because the surgical instruments with MAGS can remove rigid body linkages, and be anchored on the inner wall of the abdominal cavity only by magnetic force [4].

The magnetic surgical instruments are mostly actuated by micro-motors or internal permanent magnets (IPMs), whose output force is smaller than that of the minimally invasive surgical instruments whose power source is placed outside of the abdominal cavity [5,6]. Laparoscope only needs to perform the posture and orientation changes, and does not require undertaking the surgical tasks that require large force. Therefore, laparoscope is very suitable for adapting MAGS. In the research on magnetic surgical instruments, the magnetic actuation endoscope is the most prevalent [7,8,9,10].

That only magnetic force is used for actuation is a current trend in the research on magnetic anchored surgical instruments. Besides magnetic actuation endoscope, there are several magnetic surgical forceps or clips [11,12,13,14] that utilize magnetic force to lift up human tissues with the assistance of other surgical instruments. Numerous clinical experiments have demonstrated the practicality of these magnetic surgical instruments [15,16]. Local magnetic actuation (LMA) can also be employed to control the movement of magnetic surgical instruments [17]. An external permanent magnet (EPM) and an internal permanent magnet form a pair of magnetic gears [18]; the IPM rotates in synchronization with the EPM, serving as the power source.

Most magnetic surgical instruments are driven by motors, which are installed at the joints [19,20,21,22,23]. The magnetic surgical instruments equipped with motors also tend to have a larger volume due to the presence of the motors. And surgeons’ vision is more obstructed by the surgical instruments, making it inconvenient to observe surgical operations. For increasing the output force, motors with bigger size need to be used, which further increases the volume of magnetic surgical instruments. Manipulators of single-port surgical robots that adopt cable transmission or a continuum body structure have a smaller diameter and greater flexibility [24]. For example, the diameter of a snake-like robot for endoscopic surgery is only 3 mm [25]. Introducing the cable transmission structure in the design of minimally invasive surgical robots offers many advantages, such as simple structure, small volume, light weight and shock-absorbing capacities [26]. These features demonstrate that the cable transmission structure is well suited for driving the motion of surgical instruments.

A magnetic anchored and cable-driven endoscope provides a wide-angle view of the surgical site, thanks to the large motion range of its flexible link [27]. The flexible link is actuated by cables connected to a power source located outside the abdominal cavity. A cable-driven camera robotic system has two DoFs provided by cables [28]. For these designs, when the endoscope is rotated along the inner wall of the abdominal cavity, the cables for driving the flexible link may have a limitation on the rotational range. In order to keep the endoscope still, the force components generated by the cable tension along the inner wall of the abdominal cavity need to be balanced with the friction and the force component of the magnetic force. This would also impose limitations on the cable tension.

Combining the advantage of good flexibility of single-port surgical robots and the fact that the surgical instruments with MAGS are not constrained by the location of the incision, we propose a new mechanical structure for magnetic surgical instruments, featuring cables to transmit power from micro-motors to the manipulator. And we made a magnetic surgical forceps prototype adopting this structure. The manipulator of the magnetic surgical forceps prototype has three DoFs, with a diameter of 10 mm.

The remainder of this paper is organized as follows. Section 2 explains the structure of the magnetic anchored and cable-driven surgical forceps. Section 3 describes the control system of the manipulator, and explains the method to achieve the precise control of the manipulator. Section 4 illustrates the variation of magnetic force on magnetic anchored and cable-driven surgical forceps with distance, and verifies the control effectiveness of the manipulator. And an ex vivo simulation experiment was conducted to validate the working capability of the magnetic anchored and cable-driven surgical forceps.

## 2. Design and Manufacturing

### 2.1. System Overview

**Figure 1:**
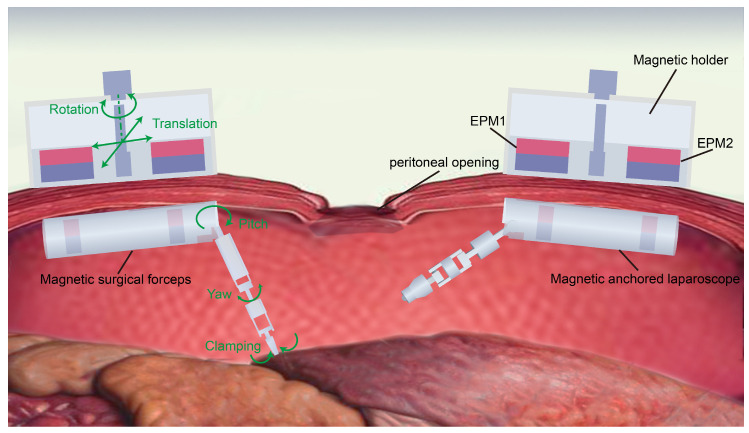
Concept of the surgery utilizing the magnetic anchored and cable-driven surgical instruments.

Figure 1 shows the appearance of the magnetic anchored and cable-driven surgical instruments during surgery. First, the magnetic anchored laparoscope is introduced into the abdominal cavity through one incision and attached onto the inner wall of the abdominal cavity through the magnetic force generated by a magnetic holder. The laparoscopic lens is aimed at the abdominal cavity incision to assist in observing the process of other magnetic surgical instruments being inserted into the patient’s abdominal cavity. After all the magnetic surgical instruments are inserted into the abdominal cavity and placed in the appropriate positions, these surgical instruments can cooperate with each other to perform the surgical operations.

The magnetic holder is positioned outside the abdominal cavity, and can be freely moved or rotated along the surface of the abdominal cavity, which in turn simultaneously drives the magnetic surgical instrument that is attached to the inner wall of the abdominal cavity, providing three DoFs, as shown in Figure 1. The manipulator of the magnetic anchored and cable-driven surgical forceps also has three DoFs, which are pitch, yaw, and clamping in sequence. As a result, the magnetic surgical forceps has a total of six DoFs.

Since the motors are not integrated into the manipulator, the manipulators of the magnetic surgical instruments have a comparable size to that of single-port surgical robots. And the magnetic surgical instruments are separated from each other during operations, which is beneficial for the cooperation.

### 2.2. Structure of the Magnetic Anchored and Cable-Driven Surgical Forceps

In the developed magnetic surgical instruments, the motors are installed near the manipulator joints, and connected to the manipulator joints through a series of mechanical parts [29,30,31]. As a result, the manipulator has a complex structure, with a large diameter and heavy weight. The diameter of the manipulator is directly related to that of the motors. To reduce the manipulator’s diameter, smaller motors are needed. For instance, the motors (SBL04) used in a modular magnetic platform [32] are 4 mm in diameter, and 5.7 mN·m in output torque.

**Figure 2:**
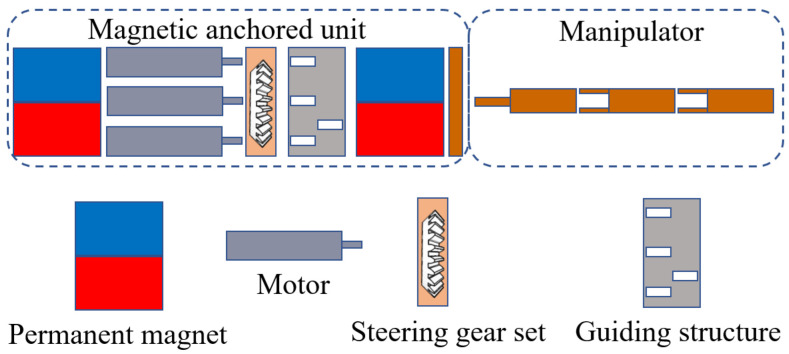
Structural diagram of the magnetic surgical instrument with cable transmission.

**Figure 3:**
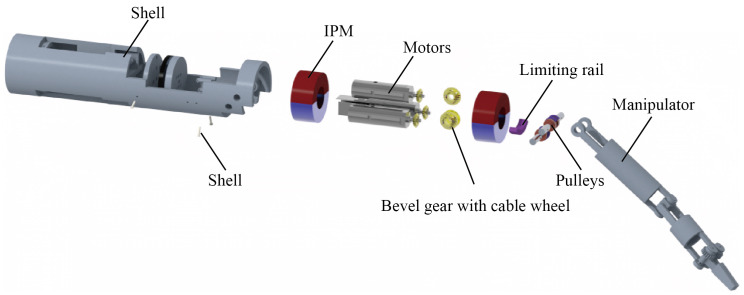
Assembly exploded view of the magnetic surgical forceps.

The structure scheme of the magnetic surgical instrument with cable transmission is illustrated in Figure 2. The structure consists of two parts: a magnetic anchored unit and a manipulator. The IPMs and the motors are both located in the magnetic anchored unit. The manipulator of the magnetic surgical instrument with cable transmission is similar with that of single-port surgical robots; the power source (motors) is separated from the manipulator. There are no motors integrated into the manipulator, so the size of the motors has no relation to the volume of the manipulator. Therefore, relatively large motors can be used to provide greater power. The assembly exploded view of the magnetic surgical forceps is shown in Figure 3. The dimensions of several magnetic surgical forceps or retractors are listed in Table 1. The manipulator of the magnetic surgical instrument designed in this paper utilizes the motors with the largest diameter of 8 mm, while the manipulator itself has the smallest diameter of 10 mm.

**Figure 4:**
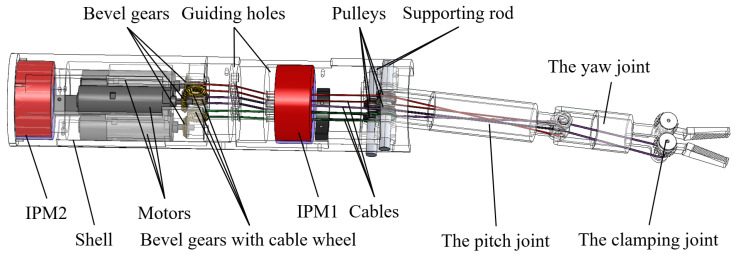
Configuration diagram of the magnetic anchored and cable-driven surgical forceps.

**Figure 5:**
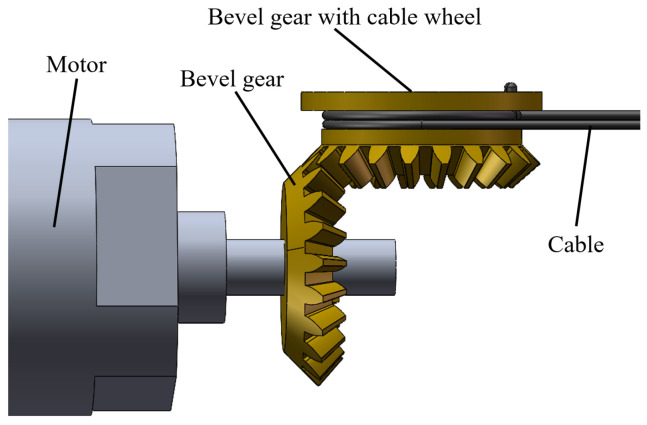
Bevel gear steering mechanism.

The motors are mounted in the magnetic anchored unit, and the motion and force from the motors are transmitted to the three joints of the manipulator by three cables, respectively. Figure 4 illustrates the specific design and components of a magnetic anchored and cable-driven surgical forceps. A pair of meshing bevel gears are responsible for shifting the direction by 90°, as shown in Figure 5. The bevel gear with cable wheel has a groove to wind cables. Another end of each cable is connected to the joint’s shaft via guiding holes.

### 2.3. Magnet Design

**Figure 6:**
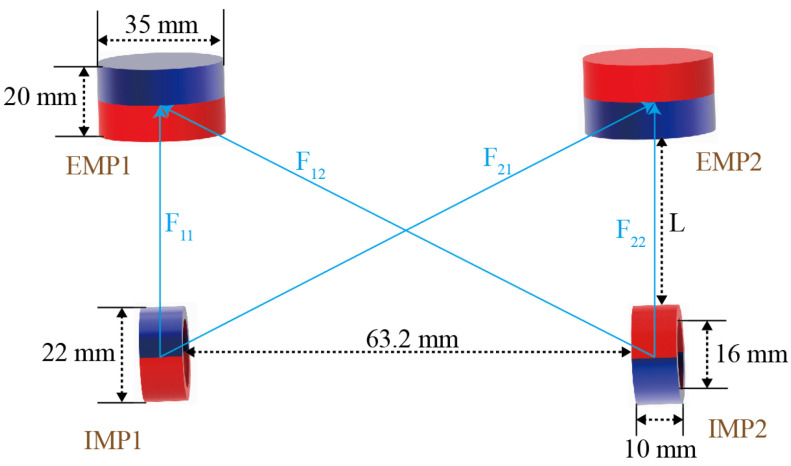
The magnetic force exerted on the magnetic surgical forceps.

Two ring-shaped IPMs, magnetized along the radial direction, are installed at opposite ends of the magnetic anchored unit. Once the magnetic surgical instrument is inserted into the abdominal cavity, the magnetic holder will attract and cause it to adhere to the inner wall of the abdominal cavity. The IPM has a length of 10 mm, an outer diameter of 22 mm and an inner diameter of 16 mm. The EPMs have a diameter of 35 mm and a length of 20 mm. Both the IPMs and EPMs are made of N52. The magnetic pole directions of the EPMs and IPMs are both in the vertical direction. The magnetic force between two EPMs in the magnetic holder and two IPMs in the magnetic surgical instrument is depicted in Figure 6. Each IPM is subjected to the magnetic force from two EPMs. So the magnetic force applied to the magnetic surgical forceps is

(1) $$F=\sum_{i=1}^{i=2}\sum_{j=1}^{j=2}F_{ij}\;\;\;\left(i=1,2;j=1,2\right)$$

where $F_{ij}$ represents the magnetic force between the *j*-th EPM and the *i*-th IPM.

The magnetic fields generated by the EPMs are calculated using the multi-dipole model. The calculation method for the magnetic field has been explained in our previous conference work [35]. In this model, the cylindrical magnet is represented by the multiple magnetic poles located on its upper and lower surfaces. The magnetic flux density at any point in space is the sum of the magnetic flux densities produced by each magnetic pole at that point.

The magnetic force can be calculated by

(2) $$F_{ij}=\int_{v}\left(M_{i}\cdot \nabla \right)B_{ij}dv$$

where $M_{i}$ is the magnetization of the *i*-th IPM, and $B_{ij}$ represents the magnetic field generated by the *j*-th EMP at the position of the *i*-th IPM.

The magnetic torque on the IPMs can be obtained by

(3) $$T=\sum_{i=1}^{i=2}\sum_{j=1}^{j=2}\int_{v}M_{i}\times B_{ij}dv\;\;\;\left(i=1,2;j=1,2\right)$$

**Figure 7:**
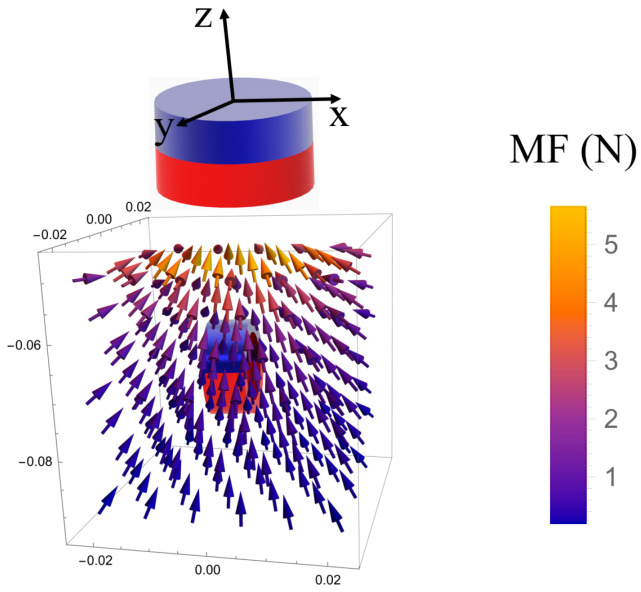
The demonstration of magnetic force field exerted on the ring-shaped IPM by the cylindrical EPM. MF: magnetic force. The unit of the coordinates in the figure is meters.

The magnetic force must be sufficient to securely attach the surgical instruments onto the inner wall of the abdominal cavity without causing harm to the human body due to excessive pressure. The magnetic force varies with the distance between the IPM and EPM, which depends on people’s skin thickness. Figure 7 depicts the magnetic force exerted on the IPM by the EPM while the centroid of the IPM is within the range of (x→(−0.02,0.02), y→(−0.02,0.02), z→(−0.05,−0.09)).

### 2.4. Prototype

**Figure 8:**
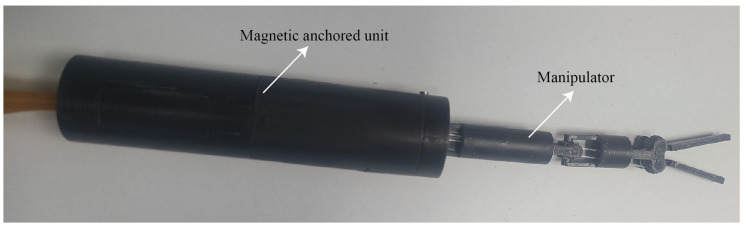
Prototype of the magnetic surgical forceps.

A prototype of the magnetic anchored and cable-driven surgical forceps was fabricated, as shown in Figure 8. Most of mechanical components were 3D-printed (printing layer thickness: 0.075 mm), which exhibits a flexural modulus of approximately 2.75 GPa and a tensile modulus of about 2.7 GPa. The bevel gears and the bevel gears with cable wheel were customized with copper, and the machining precision is 0.02 mm. The polyamide cables were selected to drive the motion of the manipulator, with a diameter of 0.2 mm and a Young’s modulus of approximately 3 GPa. The magnetic anchored unit has a diameter of 25 mm, and a length of 110 mm. The manipulator has a diameter of 10 mm. The weight of the magnetic surgical forceps is 70.3 g. The experiments show that when the distance of the magnetic surgical forceps and the magnetic holder was less than 45 mm (measured from the top of the magnetic surgical forceps to the bottom of the magnetic holder), the magnetic surgical forceps would still be attracted and held. The manipulator of the magnetic surgical forceps is driven by three stepper motors (Shenzhen ZhaoWei Machinery & Electronics Co., Ltd., Shenzhen, China, ZWPD008008-369). The motor has a diameter of 8 mm, and an output torque of 50 mN·m. Each motor independently drives one joint of the manipulator.

## 3. Control Strategy of the Manipulator

### 3.1. Motion of the Manipulator

**Figure 9:**
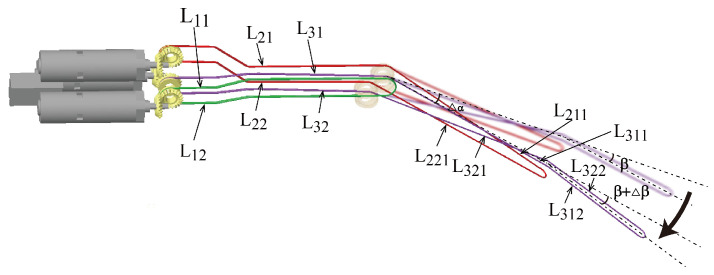
Schematic diagram of the driving cables connected to the three joints of the manipulator.

The driving cable connects the motor and the joint’s shaft, enabling the manipulator to be driven by the motors through the cables. The three driving cables are noted as $L_{1}$, $L_{2}$ and $L_{3}$. The driving cables are divided into several segments according to location, as shown in Figure 9. $L_{211}$ is the upper portion of the yaw joint-driving cable segment that passes through the pitch joint, and $L_{221}$ is the lower portion opposite $L_{211}$. Similarly, $L_{311}$ denotes the upper portion of the clamping joint-driving cable segment that passes through the pitch joint, and $L_{321}$ is the lower portion opposite $L_{311}$. While the pitch joint rotates, the yaw joint and clamping joint move together with the pitch joint due to the length changes of $L_{211}$, $L_{221}$, $L_{311}$ and $L_{321}$. While the yaw joint rotates, the clamping joint moves simultaneously with the yaw joint owing to the length changes of $L_{311}$ and $L_{321}$. To decouple the motion of the three joints, it is necessary to compensate for the length changes in the driving cables.

**Figure 10:**
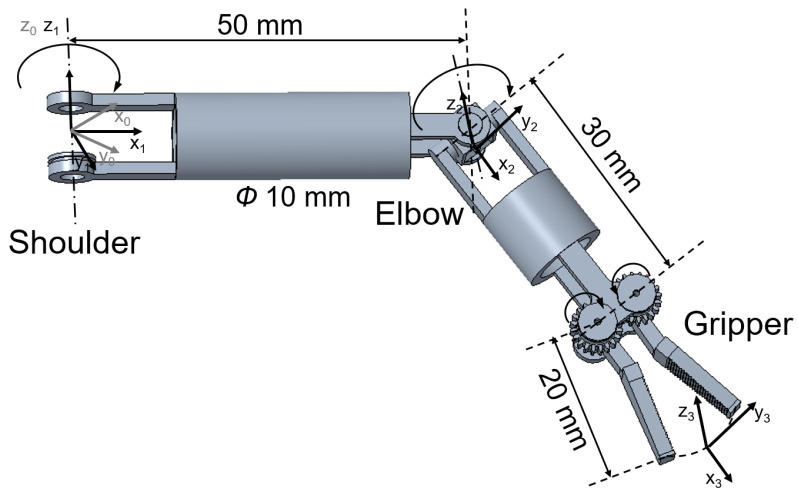
Illustration of the kinematics of the manipulator.

The three DoFs of the manipulator are actuated by three driving cables. Figure 10 illustrates the structure of the manipulator. Since no motors are integrated into the manipulator, its diameter can be reduced to 10 mm. The length of the pitch joint of the manipulator is 50 mm, that of the yaw joint is 30 mm and the length of forceps head is 20 mm. The motion range of the pitch joint is from $0^{\circ }$ to $90^{\circ }$, and that of the clamping joint is from $0^{\circ }$ to a angle exceeding $180^{\circ }$. In practice, we limit the clamping joint’s angle to $120^{\circ }$, which is sufficient to meet practical requirements. While the deflection angle of the yaw joint is larger than $40^{\circ }$ or less than $-40^{\circ }$, the control precision of the yaw joint decreases significantly. When the yaw joint’s angle is large, increased friction between the clamping joint-driving cable and yaw joint significantly contributes to the decline in control precision. So the yaw joint’s range is limited to −40–$40^{\circ }$ in practice.

**Figure 11:**
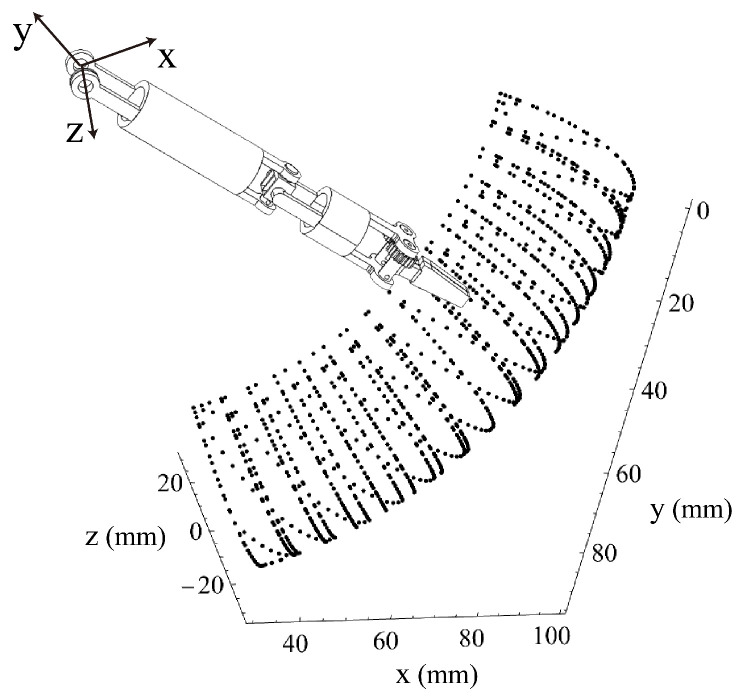
Workspace of the manipulator.

The Denavit–Hartenberg (D-H) kinematic parameters of the manipulator are listed in Table 2. Because there are only two DOFs of the manipulator for changing the orientation of the surgical forceps head, the rotation axes of these two DOFs are set to be perpendicular to each other, enabling a large operating space. Under the condition that the magnetic surgical forceps stays still, the workspace of the manipulator is depicted in Figure 11. During surgery, the magnetic surgical instrument can be moved or rotated along the inner wall of the abdominal cavity to extend the working area, allowing the tip of the manipulator to reach the lesion site and perform the surgical operations.

### 3.2. Control System of the Manipulator

**Figure 12:**
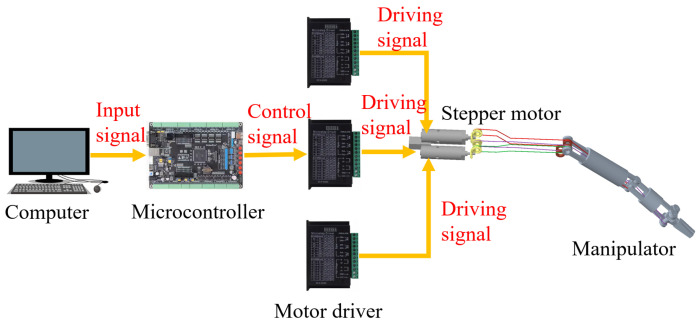
Control system framework of the manipulator.

Figure 12 shows the control system of the manipulator. The computer sends commands to the stepper motor microcontroller (STMicroelectronics, Geneva, Switzerland) via a serial port program. The microcontroller calculates the pulse count for each motor and sends the pulse signals to the stepper motor driver (TB6600), which then drives the stepper motor to move.

**Figure 13:**
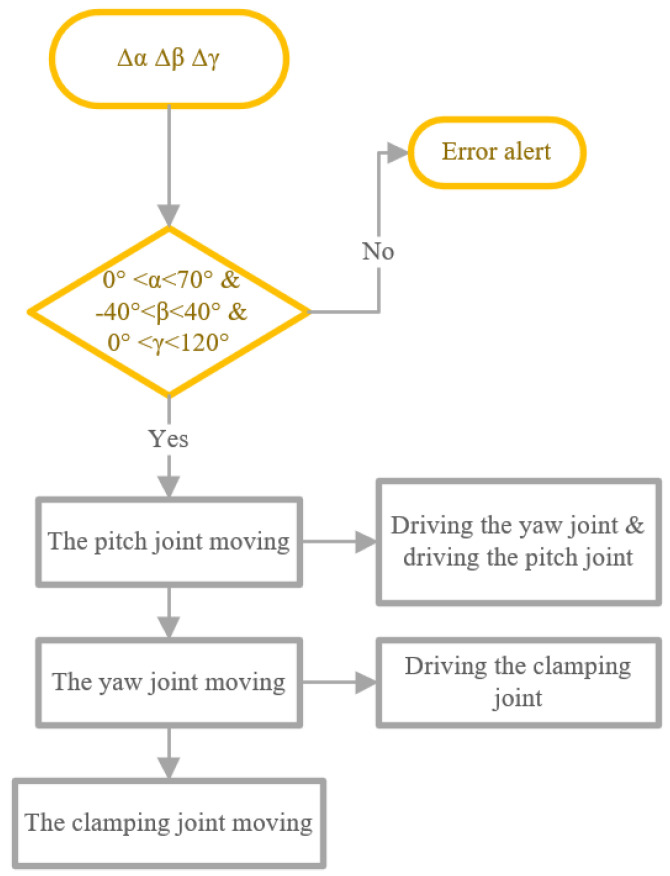
Schematic diagram of the manipulator control program.

The deflection angle of each joint is sent to the microcontroller from the computer. A manipulator-control program was embedded in the microcontroller. The structure of the program is illustrated in Figure 13. The program receives motion commands for the manipulator’s three joints. The deflection angle for the pitch joint is ▵α, for the yaw joint it is ▵β and for the clamping joint it is ▵γ. If the deflection angle of any joint exceeds its limits, the program will not drive the joints to move and will send an error alert to the computer. If the deflection angles are feasible, the program calculates and sends control signals to the motor drivers.

The pulse count for the pitch joint deflection is calculated by

(4) $$n\cdot \rho =\frac{▵\alpha *r_{1}}{r_{s}}$$

where ρ is the motor’s step angle, *n* represents the number of pulses required to drive the pitch joint by ▵α, $r_{1}$ is the radius of the pitch joint shaft and $r_{s}$ is the radius of the groove on the bevel gear.

While the pitch joint rotates, the lengths of $L_{211}$ and $L_{221}$ of the yaw joint-driving cable, and that of $L_{311}$ and $L_{321}$ of the clamping joint-driving cable, change simultaneously. This causes the yaw joint and clamping joint to undergo coupled motions. Regarding coupled motion trends, when the pitch joint rotates in the direction of increasing angle, the yaw joint rotates in a clockwise direction, and the clamping joint tends to open. To keep the yaw joint and clamping joint stable while the pitch joint rotates, the motors corresponding to the yaw joint and clamping joint are also driven simultaneously to compensate for the length changes of the driving cables.

(5) $$\begin{array}{cc} \; & n_{12}\cdot \rho =\frac{\left(L_{211}^{\prime \prime }-L_{221}^{\prime \prime }\right)-\left(L_{211}^{\prime }-L_{221}^{\prime }\right)}{4\cdot r_{s}}\\ \; & n_{13}\cdot \rho =\frac{\left(L_{311}^{\prime \prime }-L_{321}^{\prime \prime }\right)-\left(L_{311}^{\prime }-L_{321}^{\prime }\right)}{2\cdot r_{s}} \end{array}$$

where $n_{12}$ is the pulse count for the yaw joint, used to compensate the length changes of $L_{211}$ and $L_{221}$, which is caused by the motion of the pitch joint. $L_{211}^{\prime }$ and $L_{221}^{\prime }$ denote the lengths of $L_{211}$ and $L_{221}$ before the pitch joint deflection. $L_{211}^{\prime \prime }$ and $L_{221}^{\prime \prime }$ denote the lengths of $L_{211}$ and $L_{221}$ after the pitch joint deflection. The control program calculates the length of each cable segment based on the analysis of the manipulator structure. $n_{13}$ is the pulse count for the clamping joint, used to compensate for the length changes of $L_{311}$ and $L_{321}$, which is caused by the motion of the pitch joint.

While the yaw joint rotates, the lengths of $L_{311}$ and $L_{321}$ change, causing the clamping joint to undergo coupled motion. Therefore, it is necessary to compensate the length change of the clamping joint-driving cable to achieve decoupling.

(6) $$n_{23}\cdot \rho =\frac{\left(L_{311}^{\prime \prime }-L_{321}^{\prime \prime }\right)-\left(L_{311}^{\prime }-L_{321}^{\prime }\right)}{2\cdot r_{s}}$$

where $n_{23}$ is the pulse count for the clamping joint, used to compensate the length changes of $L_{311}$ and $L_{321}$, which is caused by the motion of the yaw joint.

When the joint located ahead moves, due to the length change of several cable segments, the cables for driving the joints located behind undergo coupled motions. The program achieves decoupling among the joints by compensating the length changes of several cable segments of the coupling joints, enabling independent control of the three joints of the manipulator.

## 4. Experimental Verification

### 4.1. Magnetic Attractive Force

**Figure 14:**
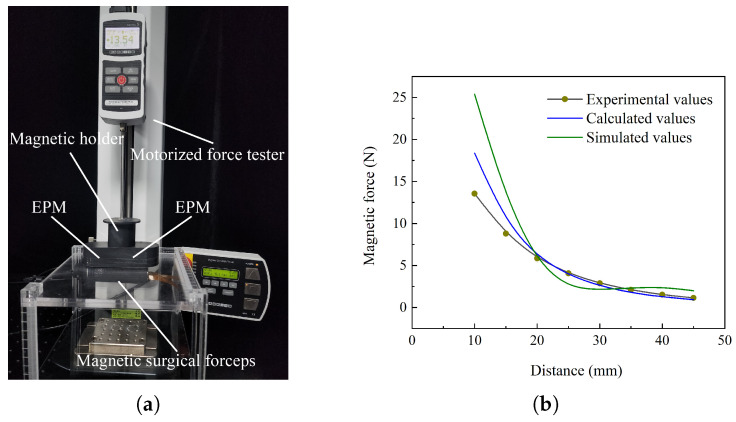
Experiment for measuring magnetic force. (**a**) Experimental setup for measuring magnetic force at different distances. (**b**) Variation of magnetic force with distance.

An experiment was conducted to measure the magnetic force exerted on the magnetic surgical forceps. The experimental platform is displayed in Figure 14a. A magnetic holder with two embedded EPMs was fixedly connected to the force gauge (M5-20, Mark-10 Corporation, Copiague, NY, USA). During the experiment, the magnetic surgical forceps was adsorbed to the underside of a 10 mm thick acrylic plate. The motorized force tester (ESM303, Mark-10 Corporation, Copiague, NY, USA) was used to drive the force gauge in vertical motion, with a movement accuracy of 0.05 mm. Initially, the motorized force tester was controlled to move the magnetic holder toward the acrylic plate. The height of the magnetic holder was then finely adjusted to make the force gauge have the maximum value. In this state, the distance between the magnetic holder and the magnetic surgical forceps was 10 mm. Subsequently, the magnetic holder was gradually raised to record the magnetic force at various distances. The experiment shows that the magnetic surgical forceps detached at a distance of 47 mm. Therefore, we measured the variation of magnetic force within the distance range of 10–45 mm.

The magnetic force exerted on the magnetic surgical forceps was simulated using Maxwell. The experimental, simulation and model calculation data are presented in Figure 14b. The model calculation data more accurately capture the variation trend of the magnetic force on the magnetic surgical forceps compared to the simulation results. The model calculation error is 1.7868, while the simulation error is 4.5224 (Root mean squared error). The model predicts a faster rate of change when the distance is less than 20 mm. Beyond 20 mm, the change rate of magnetic force calculated by the model closely aligns with the experimental measurements. The maximum error under this condition occurred at 45 mm, reaching 19.52%.

Once the magnetic surgical forceps is introduced into the abdominal cavity, it is immediately adsorbed onto the inner wall of the abdominal cavity. The magnetic surgical forceps is required to perform load-bearing tasks during operations. Therefore, the magnetic force not only needs to overcome the gravity of the magnetic surgical forceps, but also to support additional operational loads. The weight of the magnetic surgical forceps is 74.6 g. The normal thickness of the abdominal wall ranges from 20 to 40 mm [18,36]. Experimental results indicate that the magnetic force is 5.86 N at a distance of 20 mm and 1.52 N at a distance of 40 mm. Consequently, the magnetic surgical forceps can withstand vertical loads of 5.13 N and 0.79 N at distances of 20 mm and 40 mm, respectively.

### 4.2. Load Capacity of the Manipulator

**Figure 15:**
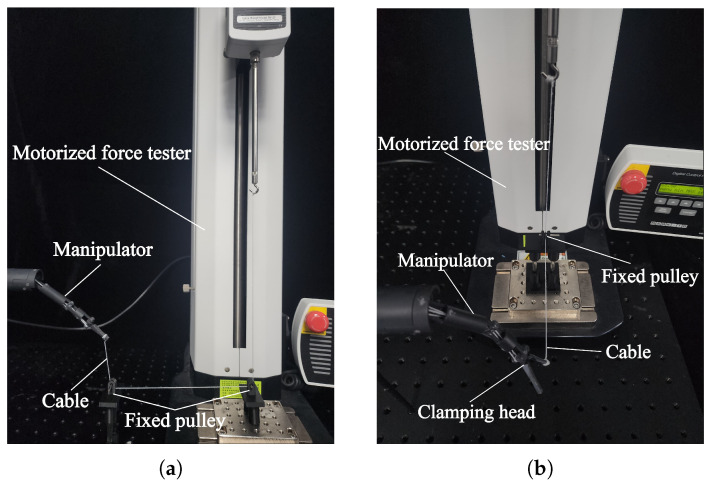
Experiments for testing the mechanical performance of the manipulator. (**a**) Experimental setup for measuring the lifting force. (**b**) Experimental setup for measuring the clamping force.

The manipulator of the magnetic surgical forceps is responsible for performing surgical operations. The load-bearing capacity is an important property for the manipulator. Two experiments were conducted to determine the maximum lifting force at the manipulator’s tip and the clamping force of the forceps, respectively. As shown in Figure 15a, One end of the cable was tied to the tip of the manipulator, while the other end was routed through two fixed pulleys and connected to the force gauge. The pitch joint was controlled to drive the manipulator to rotate counterclockwise, and the force gauge recorded an upward lifting force of 0.22 N.

Figure 15b illustrates the test bench used to determine the clamping force of the magnetic surgical forceps. During the measurement of the clamping force, when measuring the clamping force of the surgical forceps head, the surgical forceps head was positioned in an open posture. A cable was tied to one jaw of the surgical forceps head, with this jaw perpendicular to the cable. The other end of the cable was connected to the force gauge via a fixed pulley. As the magnetic surgical forceps was driven to close, there was a tendency to pull the cable, and the force gauge recorded a force of 0.75 N on one jaw of the surgical forceps head.

### 4.3. Control of the Manipulator

**Figure 16:**
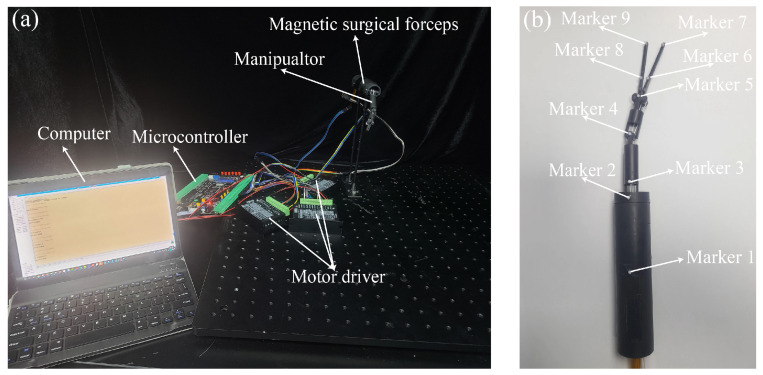
Experimental setup for capturing the motion of the manipulator. (**a**) The control platform of the manipulator. (**b**) The deployment of the markers on the magnetic surgical forceps.

The control method of the manipulator is detailed in Section 3. In this section, a series of experiments were conducted to evaluate the control performance of the manipulator’s motion. The experimental platform is shown in Figure 16. A motion-capture system (NOKOV) was employed to record the joint angles of the manipulator. The motion-capture system has a residual error of 0.15 mm. The control system of the manipulator is shown in Figure 16a. The markers attached to the manipulator are shown in Figure 16b. To facilitate the identification of markers, the length of the surgical forceps head was extended from 20 mm to 40 mm. The joint angles of the manipulator were calculated based on the coordinates of the markers.

The pitch joint was driven to rotate from 0° to 70° in increments of 10°. During the experiment, due to motion errors, when the pitch joint was driven to rotate to 70°, the pitch joint consistently reached its maximum deflection angle. So this position could not reflect the control precision, and was excluded from the motion accuracy measurements for the pitch joint. When the yaw joint rotated to an angle greater than 40°, or less than −40°, the control precision decreased significantly. So the motion range of the yaw joint is limited to −40° to 40° in practice. The yaw joint was driven to rotate from −40° to 40° in increments of 10° to assess the control accuracy during the experiment. The angle of the clamping joint refers to the angle between the two jaws of the surgical forceps. The clamping joint was driven to move from 0° to 120° in increments of 20°.

**Figure 17:**
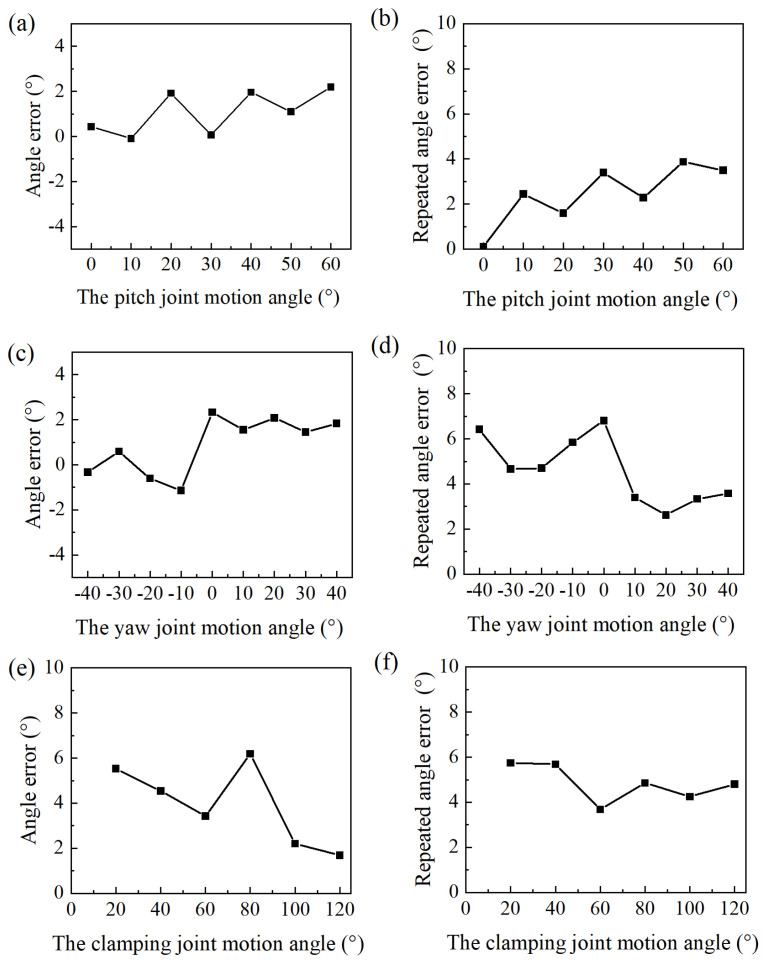
Motion error and repeatability error of joint motions. (**a**) Motion error of the pitch joint. (**b**) Repeatability error of the pitch joint. (**c**) Motion error of the yaw joint. (**d**) Repeatability error of the yaw joint. (**e**) Motion error of the clamping joint. (**f**) Repeatability error of the clamping joint.

Each set of driving experiments was repeated five times. The accuracy and repeatability of the joint motions are shown in Figure 17. The angle error shown in Figure 17 represents the deviation between the average motion angles of the joints and the target angles. Repeatability refers to the range of angle fluctuations observed across five experiments. The average error of the pitch joint motion angle was 1.51°. The angle fluctuation of the pitch joint was the largest at 50°, measuring 3.87°. The average error of the yaw joint motion angle was 1.32°. The repeatability experiment of the yaw joint motion shows an average angle fluctuation of 4.6° within the range of −40° to 40°. The angle fluctuation of the yaw joint was the largest at 0°, measuring 6.8°. The angle error of the clamping joint motion was slightly larger than that of the pitch joint or the yaw joint, measuring 3.4°, with a repeatability error of 4.83°.

### 4.4. Ex Vivo Experiment

**Figure 18:**
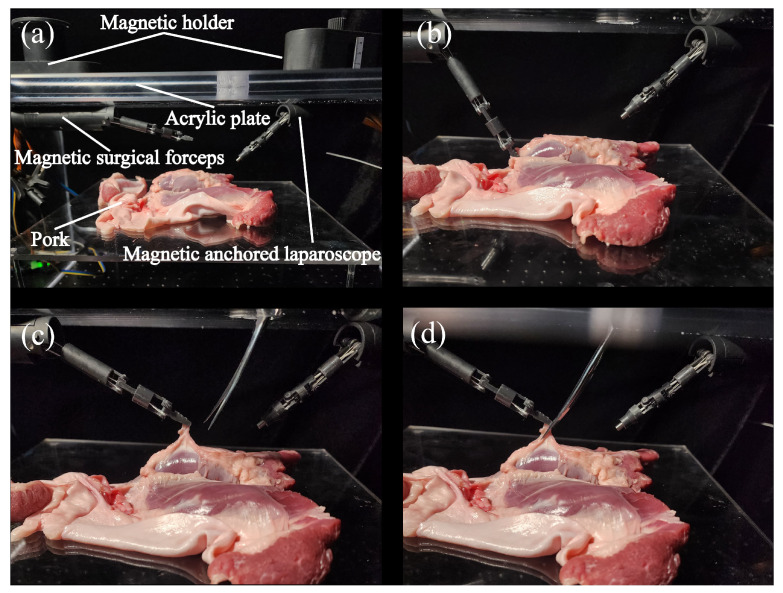
Ex vivo experiment. (**a**) The experimental platform. (**b**) The magnetic surgical forceps tips approached and held a piece of pork. (**c**) A piece of pork was picked up. (**d**) Surgical scissors cut the lifted pork.

Another laparoscopic surgery-simulation experiment was used to verify the performance of the magnetic surgical forceps. Two magnetic anchored and cable-driven surgical instruments were developed: a magnetic surgical forceps and a magnetic anchored laparoscope. Except for differences in the joint configurations of the manipulators, the structures of these two surgical instruments are generally similar, as shown in Figure 18. During surgery, once the magnetic surgical instruments are inserted into the abdominal cavity through the abdominal incision, only the wires for transmitting electricity or data remain at the incision site. This allows another surgical instrument to be used to perform the surgical operations in cooperation with the magnetic surgical instruments positioned within the abdominal cavity.

A 15 mm thick acrylic plate was employed to simulate human skin. A piece of pork was used to mimic the tissues within the human abdominal cavity, as shown in Figure 18. A magnetic anchored and cable-driven surgical forceps and a magnetic anchored and cable-driven laparoscope were adsorbed onto the underside of the acrylic plate. The lens of the magnetic anchored and cable-driven laparoscope was oriented toward the surgical site to photograph the operation process. The manipulator of the magnetic surgical forceps was driven to position the surgical forceps tip near the pork, and pick a piece of pork up, as shown in Figure 18c. Surgical scissors were introduced via the incision to cut the bit of pork that was grasped by the magnetic surgical forceps, as shown in Figure 18d. So the tissue-excision task was completed through the collaboration of the magnetic surgical instruments and a surgical scissors.

## 5. Discussion

The primary contribution of this paper is that it is the first time that cables are employed to transmit motion and force from micro-motors to the manipulator. By employing a cable-driven mechanism, we aim to minimize the diameter of the manipulator while utilizing relatively larger motors. Reducing volume and enhancing output force are important targets that the research of magnetic surgical instruments has been pursuing. The magnetic actuated endoscope has been well developed thanks to it having no requirements on output torque. Several flexible and compact magnetic actuated endoscopes that are driven only by magnetic force have been developed [7,10,37]. Pure magnetic actuation remains a highly popular research topic and development direction in the field now. Except for magnetic actuated endoscopes, the research on other magnetic surgical instruments, such as magnetic surgical forceps and magnetic retractors, has faced challenges in balancing volume and output force. Several attempts have been made to apply pure magnetic actuation to the development of these surgical instruments [38,39,40]. However, when implementing pure magnetic actuation in the design of such surgical instruments, magnetic interference issues must be effectively addressed. When multiple embedded IPMs are used as the power source to drive the magnetic surgical instrument to perform surgical operations, the distances between the IPMs needs to be kept at a certain level for independent motion control. To avoid excessive volume related to the abdominal cavity, not too many IPMs should be installed in the body of the magnetic surgical instrument. This also has limitations with respect to the DoFs. In addition, the pure magnetic actuation does not demonstrate a significant advantage over micro-motors in terms of output force [3]. The developed magnetic surgical instruments that utilize motors as the power source have the motors installed near the joints. The size of the motors directly influences the dimensions of the manipulator. The manipulator of the modular magnetic platform designed by Tortora [41] has a small diameter of 12 mm. However, the small motors (diameter: 4 mm) used in the modular magnetic platform provide a limited torque of 5.7 mN·m. Using larger motors can enhance the output torque, but it also results in an increased diameter of the manipulator. For example, the manipulator of the magnetic surgical forceps design by Fu [31] has a diameter of 20 mm. The motor has a diameter of 6 mm, with an output power of 0.3 W. Similarly, the manipulator of the dexterous miniature robot designed by Doleynik [34,42] has a diameter of around 17 mm. The manipulator of the single-port surgical robot designed by Yeung [43] has a diameter of 15.8 mm. The manipulators with larger diameters are more prone to mutual interference and obstruct the surgeon’s field of view to a greater extent. In our magnetic surgical forceps, micro-motors also serve as the power source. But the motors are separated from the manipulator by cables. Compared to the developed magnetic surgical forceps or retractors listed in Table 1, the magnetic anchored and cable-driven surgical forceps adopted larger micro-motors, and the manipulator was designed and manufactured with a minimum diameter of 10 mm.

Cable-driven actuation is a mechanism that transmits motion and force from a power source to a remote site via flexible tubes or pulleys. The tendon-sheath mechanism, where cables pass through flexible tubes, is widely used in minimally invasive surgical robots [2,6]. This mechanism exhibits high friction between the cable and the sheath. For minimally invasive surgical robots, the servo motors typically provide sufficient torque, making the high friction not a problem. However, the micro-motors used in magnetic surgical instruments provide significantly lower torque compared to the servo motors employed in minimally invasive surgical robots. Implementing the tendon-sheath structure in magnetic surgical instruments would lead to a significant power loss due to the friction between the cable and the sheath. Therefore, the tendon-sheath mechanism is unsuitable for magnetic surgical instruments.

We adopt guiding holes and pulleys to transmit motion or force from motors to the manipulator joints. The pitch joint motion changes the lengths of the driving cables for both the yaw joint and clamping joint, and the yaw joint motion changes the length of the clamping joint-driving cable. This results in motion coupling between the joints [44], leading to a degradation of the motion accuracy. Magnetic surgical instruments have a small volume, making it difficult to install a decoupling mechanism [45,46,47]. We analyzed the length changes of the driving cables connected to coupling joints of the manipulator, and achieved decoupling by compensating for these length changes, known as active compensation.

This structure stems from the idea of separating the power source from the manipulator, that is similar to the traditional minimally invasive surgical robots, but is rarely adopted in magnetic surgical instruments. This design can not only be applied to the magnetic surgical forceps described above, but can also be applied to other magnetic surgical instruments, by simply modifying the design of the end-effector of the manipulator. A magnetic anchored and cable-driven laparoscope that is not detailed in this text was used in Section 4.

Several magnetic surgical instruments can be introduced into the abdominal cavity through one incision in sequence. The magnetic surgical instruments with different functions can cooperate with each other to complete the surgical procedure. After the surgical instruments are inserted into the abdominal cavity, there are only wires for transmitting the data or electricity to be left at the incision site. As a result, another surgical instrument could join the surgical operations via the incision. Alternatively, a single magnetic surgical instrument could be used to assist the single-port surgical robot in performing surgical operations as needed. A laparoscope could be employed to assist in capturing images of the surgical site, providing an additional perspective. The manipulators of these surgical instruments have a structure and size similar to that of minimally invasive surgical robots with cable transmission, which facilitates better collaboration between different surgical instruments.

## 6. Conclusions

A magnetic surgical forceps with a novel structure is proposed in this paper. This design is characterized by utilizing cables to transmit the motion and force from micro-motors to the manipulator. Compared to integrating motors directly into the manipulator, the cable-driven actuation enables a simpler and more compact manipulator structure. A magnetic anchored and cable-driven surgical forceps prototype was manufactured. And the diameter of the manipulator is limited to 10 mm while employing the motors with a diameter of 8 mm. The magnetic surgical forceps have six DoFs, and the three DoFs of the manipulator are driven by three micro-motors separately. The manipulator control system was experimentally validated, and an ex vivo experiment preliminarily verified the practicability of the magnetic anchored and cable-driven surgical forceps. In future work, more adequate experiments need to be conducted to verify the working ability of the magnetic surgical forceps. Additionally, structural optimization can be pursued to further reduce the volume and minimize force loss during transmission, and the design of the external magnetic holder should be refined and encapsulated to make a highly finished product.

## Figures and Tables

**Table 1:** Parameters of several magnetic surgical instruments.

Surgical Instrument	Magnetic Anchored Unit		Manipulator		Motor Diameter (mm)	DoF of Manipulator
	Length (mm)	Diameter (mm)	Length (mm)	Diameter (mm)		
Feng [31]			110	20	6	3
Tortora [33]	186	17	95	12	4	4
Oleynikov [34]	80	26	53	26	6	4
Valdastri [18]	154	12.5			No motor	1
This paper	110	25	100	10	8	3

**Table 2:** D-H parameters.

Joint	$\alpha_{i-1}{(}^{\circ })$	$a_{i-1}$ (mm)	$d_{i}$ (mm)	$\theta_{i}\;{(}^{\circ })$
1	0	0	0	0∼70
2	90	50	0	−40∼40
3	0	50	0	0

## Data Availability

The original contributions presented in the study are included in the article, further inquiries can be directed to the corresponding author.
